# A mural nodule of anaplastic carcinoma with sarcomatoid differentiation in a background of ovarian borderline mucinous cystadenoma

**DOI:** 10.3332/ecancer.2023.1557

**Published:** 2023-06-05

**Authors:** Kasiemobi Eberechukwu Uchime, Oludolapo Andrea Akinjo, Nicholas Awodele Awolola, Ephraim Ohazurike, Adekunbiola Aina Banjo, Igbokwe Uchechi

**Affiliations:** 1 Department of Anatomic and Molecular Pathology, Lagos University Teaching Hospital, Lagos 102215, Lagos State, Nigeria; 2 Department of Anatomic Pathology and Forensic Medicine, Afe Babalola University Ado-Ekiti (ABUAD) Multi-system Hospital, Ado-Ekiti 360102, Ekiti state, Nigeria.; 3 College of Medicine, University of Lagos, Lagos 101014, Lagos State, Nigeria; 4 Department of Obstetrics and Gynecology, Lagos University Teaching Hospital, Lagos 102215, Lagos State, Nigeria; 5 Department of Anatomic Pathology, Queens Hospital, Romford RM7 0AG, Essex, UK

**Keywords:** ovarian tumour, mucinous cystic neoplasms, mural nodules, anaplastic carcinoma, sarcomatoid differentiation, case report

## Abstract

Ovarian mucinous cystic tumours with mural nodules are rare tumours of the ovary that are often missed out during diagnosis. They are classified under the ovarian mucinous surface epithelial-stromal tumours. These mural nodules can be sarcoma-like (benign), anaplastic carcinoma, sarcomas, or mixed malignant (carcinosarcoma). However, very few cases of anaplastic malignant mural nodules have been reported. Here, we present a case of a borderline ovarian mucinous cystadenoma with anaplastic mural nodule that has sarcomatoid differentiation, in a 39-year-old woman who presented with a 1-year history of progressive abdominal swelling and pain. There were intraoperative findings of huge right ovarian cystic tumour with omental and umbilical deposits. Differential diagnosis of possible germ cell tumours, vascular tumours, melanoma, sarcoma and sarcoma-like nodules were ruled out with routine histology (Haematoxylin & Eosin), histochemical (reticulin) and immunohistochemical stains (CK AE1/3+, CD30+, AFP−, HCG−, EMA−, S100 protein−, CD31−, and CD34−) and the final diagnosis of a mural nodule of anaplastic carcinoma with sarcomatoid differentiation in a borderline ovarian mucinous cystadenoma established. Unfortunately, due to the aggressive nature of the tumour and disease progression, the patient passed on a few months after the surgery. This rare tumour, especially the ones with anaplastic carcinoma or mixed tumours, usually has an aggressive clinical course with most patients presenting late when the disease is advanced with poor clinical outcomes as is seen with the index patient. A high index of suspicion of this tumour with early detection and a multidisciplinary approach to its management is advised.

## Introduction

Mucinous cystic tumours of the ovary with mural nodules are well-described tumours that are rare and often missed out during diagnosis [1, 2]. They are defined as the presence of one or more discrete nodules in the wall of a mucinous cystic ovarian neoplasm that may be benign, borderline or malignant [1]. They are classified under the mucinous surface epithelial–stromal tumours of the ovary [1, 2]. They are subdivided into sarcoma-like mural nodules, anaplastic carcinoma, sarcomas, or mixed nodules [1, 2]. The presence of sarcoma-like nodules is not of any prognostic significance. However, nodules of anaplastic carcinoma, sarcoma or mixed nodules (i.e., sarcoma with anaplastic carcinoma or carcinosarcoma) have a poorer clinical outcome [1].

Nodules of anaplastic carcinoma almost always occur in the background of borderline or malignant mucinous ovarian tumours [2]. These nodules are usually yellow, pink or red with areas of haemorrhage or necrosis on gross examination [2]. Microscopically, they are composed of large, round or spindle-shaped cells with abundant eosinophilic cytoplasm and high-grade malignant nuclei [2]. They usually show a strong cytoplasmic immunostain for cytokeratin [2]. Foci of anaplastic carcinoma are found more often in advanced-stage tumour [2]. However, they are not necessarily associated with adverse clinical outcomes when they are confined in intact Stage 1A tumours [2]. According to Zhang *et al* [3] prior to their report in 2013, less than 80 cases of ovarian mucinous cystic tumours with anaplastic carcinoma have been reported in the last 50 years. Methods of establishing an accurate diagnosis of this tumour are limited as the tumour does not have definitive identifying molecular markers. A case of anaplastic carcinoma mural nodule with sarcomatoid differentiation in a background of ovarian borderline intestinal type mucinous cystic tumour of the ovary is presented in this literature. Ancillary techniques including special stains and immunohistochemistry were employed to distinguish the histopathologic features from other possible differentials and to establish the diagnosis.

## Case presentation

### Clinical findings

A 39-year-old woman, para 2 (3 Alive), who presented to the gynecological emergency unit of the Lagos University Teaching Hospital, Lagos, Nigeria, with a 1-year history of progressive abdominal swelling, a 2-week history of worsening abdominal pain, and a 5-day history of low-grade fever. She had an earlier abdominopelvic ultrasound scan done in a private health facility that revealed a right ovarian cyst. An earlier ultrasound-guided percutaneous drainage of the ovarian cyst, performed at a peripheral hospital, yielded an amber-colored fluid that was sent for cytologic analysis. The cytology report was negative for malignant cells.

On physical examination, she was found to be mildly pale, febrile (*T* = 37.6°C) and anicteric. Her abdomen was distended. There was abdominal guarding and tenderness with an epigastric bulge noted.

Preoperative complete blood count revealed a haemoglobin concentration of 7.6 g/dl, white blood cell count of 14.29 × 10^9^/L, platelet count was 543 × 10^9^/L. Serum electrolyte, urea and creatinine were normal. Liver function test was normal apart from elevated gamma glutaryl transferase at 121 U/L. The serum CA-125 was elevated at 66.5 U/mL. The other tumour markers requested (Carcinoembryonic antigen (CEA), Alpha-fetoprotein (AFP), and quantitative beta-human chorionic gonadotropin (HCG)) were normal. Pre-operative chest X-ray was normal.

Preoperative abdominopelvic computed tomography (CT) scan revealed a huge heterogeneous, majorly cystic, septated and multiloculated mass in the right adnexum measuring 15.6 × 16.8 × 25.5 cm in anteroposterior, transverse and coronal sections. There was also mild fluid collection noted in the pelvis. The uterus and other intraabdominal structures were normal.

A presumptive diagnosis of right ovarian tumour (CT diagnosis), most likely malignant with possible intra-abdominal sepsis was made.

She was immediately commenced on broad-spectrum intravenous antibiotics (Ceftriaxone and Metronidazole) and later extensively counseled on the above presumptive diagnosis and management options including the possibility of a staging laparotomy for suspected ovarian malignancy to which she agreed. A written informed consent for the surgery was then obtained after the counseling.

She was then scheduled for an emergency exploratory laparotomy in view of the acute abdomen, huge right ovarian tumour as well as clinical and haematologic evidence of ongoing sepsis. She later had the emergency exploratory laparotomy with Total Abdominal Hysterectomy and Bilateral Salpingo-oophorectomy with Omentectomy (TAH & BSO). The important surgical findings include haemorrhagic ascitic fluid ( ~ 1.5 L), a huge punctured right cystic ovary measuring 20 × 16 × 15 cm, an omental nodule, a subcutaneous periumbilical nodule, and moderate adhesions between the bowels, omentum, right ovary and the anterior abdominal wall. The excised TAH & BSO with omentectomy samples and an excised periumbilical subcutaneous nodule were sent for histopathologic evaluation.

However, about 6 days post-op (post-initial emergency laparotomy), the patient became anemic and febrile. Imaging studies done on the patient suggested an intrabdominal abscess collection for which she subsequently had another emergency abdominopelvic re-exploration for the evacuation of the abdominal abscess. At this second emergency surgery, she had about 3 L of haemoperitoneum with numerous ragged haemorrhagic amorphous tumour seedlings extensively infiltrating the majority of the abdominal cavity and encasing the small bowel loops and friable mesentery. There were dense adhesions between the bowel loops, and a dilated small bowel. She was then admitted to the intensive care unit for post-operative care in view of her poor clinical state post-surgery. The patient did not receive any chemotherapy. Unfortunately, the patient passed on a few months after the second emergency surgery due to the progression of the disease.

### Pathologic findings

The specimen received from the first emergency surgery (laparotomy with TAH & BSO and omentectomy) included the uterus with its adnexae, an omental tissue and a soft tissue labeled ‘ periumbilical subcutaneous mass’. There was a huge right ovarian mass that weighed 3 kg and measured 26 × 20 × 15 cm. The surface of the ovarian mass was smooth with areas of fibrinohemorrhagic exudates. The cut section of the ovarian mass revealed a mutiloculated cystic cavity containing about 3 L of mucoid fluid. Within the lumen of the cyst were multiple areas of yellowish mucoid soft-to-firm solid excrescences. Each of these excrescences measured about 0.2 × 0.2 × 0.2 cm. Their cut surfaces were yellowish and mucoid.

The serosal surface of the uterus and its adnexae were haemorrhagic. The uterine adnexae were matted to the uterus. However, there was no obvious mass seen within the uterus, uterine adnexae and cervix grossly.

There was a haemorrhagic, dark-greyish solid mass with an infiltrative border, measuring 8 × 6 × 4 cm, within the submitted omental tissue.

**Figure 1.**
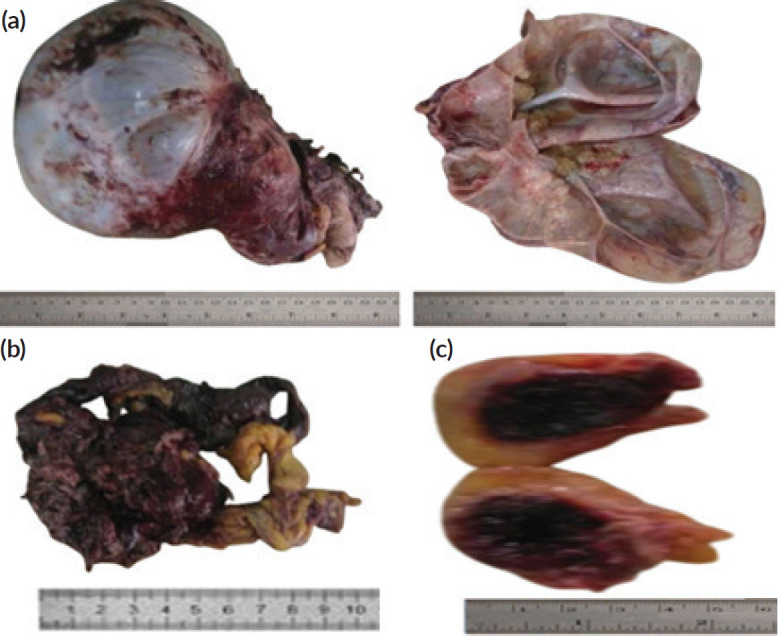
Gross picture of the cystic ovarian mass, omental deposit and umbilical subcutaneous nodular deposit. (a): Gross picture of the cystic ovarian mass. (b): Gross picture of the omental deposit. (c): Gross picture of the umbilical subcutaneous nodular deposits.

The specimen tagged ‘subcutaneous mass’ (obtained from the anterior abdominal periumbilical region) was a pendunculated fibrofatty haemorrhagic tissue that measured 4 × 3 × 3 cm. Its cut section revealed a well-circumscribed, soft, haemorrhagic mass that measured 2.5 x 2.0 x 1.0cm. The gross pictures of all the received tissue biopsies are as shown in Figure 1.

**Figure 2.**
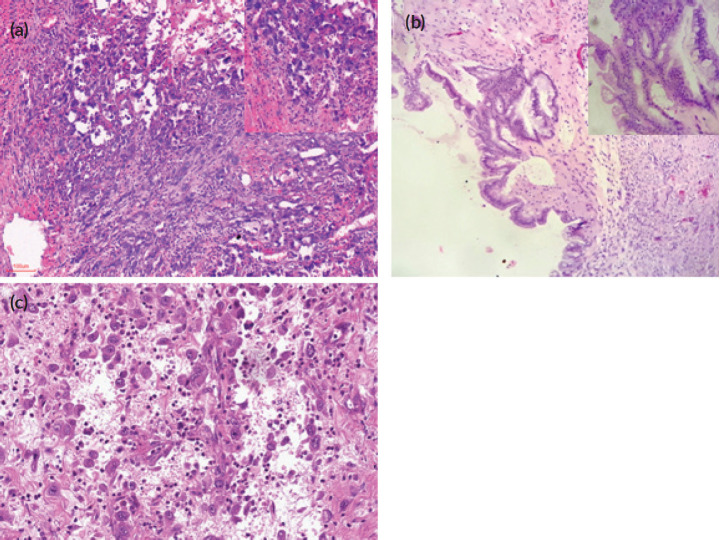
Photomicrographs (H&E) stains of the ovarian anaplastic mural nodule and omental deposit. (a) H&E x100 and x400 (inset) magnification of the ovarian malignant anaplastic mural nodule. (b) H&E x100 and x400 (inset) magnification of focal areas of ovarian borderline mucinous cystic tumour. (c) H&E x 200 magnification of the omental deposit.

Microscopically, the major parts of the wall of the right ovarian cyst were lined by a single layer of columnar mucin-secreting, intestinal-type epithelium. However, there were focal areas of the wall that were lined by two to three-layered stratified atypical cells, some having goblet cells. There were adjacent areas of a cystically degenerated tumour nodule in which atypical spindled- to-rhabdoid-to-epithelioid pleomorphic large-sized tumour cells were irregularly distributed in a relatively abundant and very loose highly vascularised/haemorrhagic stroma, with a mild mixed inflammatory cell infiltrates. The large-sized tumour cells were markedly atypical with abundant eosinophilic cytoplasm and mildly hyperchromatic nuclei that often have large prominent nucleoli. There were numerous mitoses including atypical forms. Occasional osteoclast-like multinucleated tumour cells are seen. The cells were irregularly distributed individually and in small clusters without definite secondary structures, no glandular differentiation, and no evidence of cytoplasmic keratinisation. Some of these atypical cells were distributed in a peritheliomatous pattern. Many plump atypical spindle cells that appear to be fibroblastic, albeit non-monotonous, were also noted. There were no areas of haematogenous or lymphovascular invasion. There were no teratomatous germ cell tumour cells seen. Sections of the omental and subcutaneous masses showed deposits of similar malignant tumour cells. Photomicrographs of the mural nodule, ovarian cystic tumour and omental deposits are as shown in Figure 2.

**Figure 3.**
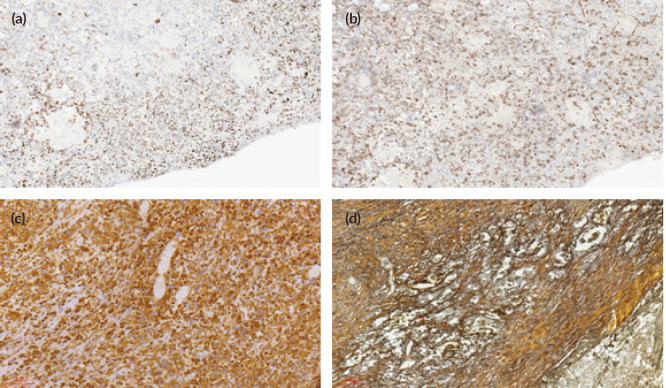
Photomicrographs of the immunohistochemical stains (CK AE1/3, CD 30, vimentin), and histochemical stains (reticulin) of the tumour cells. (a): Immunohistochemical staining (×40 magnification) shows reactivity for CK AE1/3. (b): Immunohistochemical staining (×40 magnification) shows focal reactivity for CD30. (c): Immunohistochemical staining (×40 magnification) shows reactivity for vimentin. (d): Reticulin histochemical staining (×40 magnification) stains reactive stromal cells.

On immunohistochemistry, there were a fairly uniform expression of CD30 and vimentin and variable weak-to-strong expression of broad-spectrum cytokeratins (AE1/3). The tumour cells were completely negative with EMA, HCG, CD31, CD34, S100 protein and AFP. The surrounding stroma cells stain positively with reticulin histochemical stain. The photomicrographs of the cytokeratin AE1/3, CD30, vimentin immunostains and reticulin stains are as shown in Figure 3.

The histologic features are those of a rare but well-defined phenomenon of a mural nodule of anaplastic carcinoma with sarcomatoid differentiation in a borderline mucinous cystic ovarian tumour.

## Discussion

Ovarian tumours with mural nodules are well-described but very rare entities. Prat and Scully [4, 5], were the first people to describe these mural nodules in mucinous cystic tumours. Many of the reported mural nodules, like the index case, have background ovarian mucinous cystic tumour, although few background serous cystic tumours have been reported [6–11]. 

Many histologic types of mural nodules have been described in different reports [4–11]. This ranged from reactive non-neoplastic nodules to neoplastic ones [4–11]. The neoplastic nodules described were either epithelial, non-epithelial or mixed [4–11]. Furthermore, the nodules were either benign or malignant [4–11]. The term sarcoma-like mural nodule has been discouraged for use since a detailed histologic evaluation, special stains and immunohistochemical analysis can help confirm the non-epithelial characteristics of these nodules [12].

The aetiopathogenesis of these mural nodules is uncertain. Mural nodules have also been associated with other mucinous non-ovarian cystic neoplasm, including that of pancreas and gall bladder [13–16]. A common finding in all these mucinous cystic neoplasms with mural nodules is the expression of granulocyte colony-stimulating factor [17, 18]. This suggests that these mural nodules may be derived from possible pluripotent cells in the stroma of the mucinous cystic nodules. Other studies advocate ‘collision tumours’ as the possible explanation for the presence of the mural nodules [19]. van denBerg *et al* [19] employed the use of molecular biological techniques to analyse three cases of pancreatic cystic mucinous neoplasms with sarcomatous stroma and discovered that the sarcomatous components were clonally different from each other. 

A study by Yamazaki *et al* [12] showed a case of a mural nodule of anaplastic spindle cell carcinoma arising in an ovarian mucinous cystic tumour of borderline malignancy in a patient with a background previous history of mature cystic teratoma. Yamazaki *et al* [12] put forward the possible malignant transformation of the teratoma as an explanation of the source of the malignant mural nodule. Richardson *et al* [20] also reported a similar malignant transformation from a mature cystic teratoma of the ovary. However, there was no history of any previous ovarian cystic teratoma in the case presented here.

It is important to differentiate mural nodules of anaplastic carcinoma and true sarcoma from prognostically favourable sarcoma-like nodules. Chan *et al* [21] employed the use of immunohistochemistry including cytokeratin and CEA to establish a case of a mural nodule of anaplastic carcinoma in a background of mucinous ovarian cystadenoma. In the index case, the tumour stained positively for pancytokeratin (CK AE1/3), thus ruling out sarcoma-like nodules and true sarcomas which do not usually stain positively with pancytokeratin [2, 22].

The result of the immunohistochemistry and histologic morphology of tumour cells of the index case ruled out differentials of yolk sac tumour (AFP−), sarcoma-like mural nodule (pankeratin AE1/3+), sarcoma (pankeratin AE1/3+, non-monotonous infiltration of atypical spindle cells), choriocarcinoma (HCG−), angiosarcoma (CD31−, CD34−), and melanoma (S100 protein−). The likelihood of embryonal carcinoma was rule out due to the non-conforming histologic picture (the tumour cells in embryonal carcinoma are usually crowded with indistinct cell borders and overlapping nuclei), even though the tumour cells stained positive for CD30 and broad spectrum cytokeratin AE1/3. It is not unusual to have anaplastic mural nodules stain positively with vimentin and reticulin [23]. This only shows that there is a sarcomatoid differentiation (sarcomatoid carcinoma) of the anaplastic carcinoma and not a true sarcoma [23]. In the index case, the tumour cells are both positive for cytokeratin AE1/3 and vimentin indicative of a carcinoma with sarcomatoid differentiation. More so, vimentin and reticulin are non-specific mesenchymal stains. In addition, the presence of a stromal infiltration by monotonous atypical spindle cells is required to make a diagnosis of the sarcomatous mural nodule, and this diagnostic histologic picture was not seen in the index case. More so, carcinosarcomatous mixed mural nodules have distinct carcinomatous and sarcomatous components and they are usually seen when the carcinomatous component is not mucinous, unlike our index case.

International Federation of Gynecology and Obstetrics (FIGO) Stage T1a (tumours limited to one ovary; capsule intact, no tumour on ovarian surface, no malignant cells in ascites or peritoneal washings) is considered a favourable outcome in patients that have ovarian mucinous neoplasm with malignant mural nodules [2]. However, the index case had a poor prognosis with International FIGO Stage T3, since there was infiltration of the omentum and periumbilical subcutaneous tissue by the malignant nodules. The clinical outcome of the patient in the index case was poor with rapid health deterioration culminating in her final demise within a couple of months.

To summarise, a rare case of an anaplastic carcinoma mural nodule with sarcomatoid differentiation in a borderline mucinous ovarian tumour is described in this report. Histopathologic morphology with ancillary techniques including special stains and immunohistochemistry were used to establish the diagnosis.

## Conclusion

Ovarian tumours with mural nodules are rare and are often missed during diagnosis [1, 2]. Few mural nodules that are anaplastic tumours or mixed nodules in a background of borderline mucinous cystic ovarian tumour, just like in the index case, have been reported. The prognosis of cases of mural nodules that are anaplastic or sarcomatous is poor, unlike the sarcoma-like mural nodules that have no prognostic significance [2]. We report a case of borderline intestinal-type mucinous cystic ovarian tumour with anaplastic carcinoma mural nodules that have sarcomatoid differentiation. The index patient had a FIGO stage T3, with an aggressive clinical outcome. Special stains (reticulin (+)) and immunohistochemistry (including vimentin (+), CD 30 (+), cytokeratin AE1/3 (+), EMA(−), HCG (−), CD31 (−), CD34(−), S100 protein (−) and AFP (−)) were used to establish the diagnosis and rule out differentials. More definitive molecular markers are required to enable an accurate diagnosis of this tumour. A high index of suspicion of this tumour with early detection and a multidisciplinary approach to its management is also advised.

## Informed consent statement

An informed consent for the publication of this article in a journal, including the anonymous dissemination of the patient’s clinical information for educational/medical research purpose, was obtained from the diseased patient’s next-of-kin.

## Conflicts of interest statement and financial declaration

The authors of this article have no funding or conflict of interest to disclose.

## References

[1] Nucci MR, Olivia E, Longacre TA, Gilks CB (2009). Surface epithelial stromal tumors of the ovary. Gynaecological Pathology.

[2] Travassoli FA, Devilee P (2003). World Health Organisation Classification of Tumours of the Breast and Female Genital Organs.

[3] Zhang Y, Yuan Z, Sun K (2017). Ultrasonic and pathological characteristics of ovarian mucinous cystic tumors with malignant mural nodules : two case report. Medicine.

[4] Prat J, Scully RE (1979). Sarcoma in ovarian mucinous tumours; a report of two cases. Cancer.

[5] Prat J, Scully RE (1979). Ovarian mucinous tumours with sarcoma-like mural nodules. A report of seven cases. Cancer.

[6] Lifschitz-Mercer B, Dgani R, Jacob N (1990). Ovarian mucinous cystadenoma with leiomyomatous mural nodule. Int J Gynecol Pathol.

[7] Baergen RN, Rutgers JL (1994). Mural nodules in common epithelial tumors of the ovary. Int J Gynecol Pathol.

[8] Prat J, Young RH, Sully RE (1982). Ovarian mucinous tumor with foci of anaplastic carcinoma. Cancer.

[9] Bagué S, Rodríguez IM, Prat J (2002). Sarcoma-like mural nodules in mucinous cystic tumors of the ovary revisited: a clinicopathologic analysis of 10 additional cases. Am J Surg Pathol.

[10] Gungor T, Altinkaya SO, Akbay S (2010). Malignant mural nodules associated with serous ovarian tumor of borderline malignancy: a case report and literature review. Arch Gynecol Obstet.

[11] Huang TY, Chen JT, Ho WL (2005). Ovarian serous cystadenoma with mural nodules of genital rhabdomyoma. Hum Pathol.

[12] Yamazaki H, Matsuzawa A, Shoda T (2013). Ovarian mucinous cystic tumor of borderline malignancy with a mural nodule of anaplastic spindle cell carcinoma: a case report. J Ovarian Res.

[13] Wenig BM, Albores-Saavedra J, Buetow PC (1997). Pancreatic mucinous cystic neoplasm with sarcomatous stroma: a report of three cases. Am J Surg Pathol.

[14] Pan ZG, Wang B (2007). Anaplastic carcinoma of the pancreas associated with a mucinous cystic adenocarcinoma. A case report and review of the literature. JOP.

[15] Michael W, Deniz G, Gil A (2013). Pancreatic mucinous cystic neoplasm with sarcomatous stroma metastasizing to liver: a case report and review of literature. World J Surg Oncol.

[16] Mizuno T, Eimoto T, Tada T (1999). Mucinous tumor of the gallbladder with a separate nodule of anaplastic carcinoma. Arch Pathol Lab Med.

[17] Park TC, Lee HN, Shin OR (2012). A case of a borderline mucinous tumor of the ovary with sarcoma-like mural nodules producing granulocyte colony-stimulating factor. Eur J Gynaecol Oncol.

[18] Ishiwata I, Ishiwata C, Soma M (1988). Establishment and characterization of a human ovarian anaplastic carcinoma cell line. Gynecol Oncol.

[19] van denBerg W, Tascilar M, Offerhaus GJ (2000). Pancreatic mucinous cystic neoplasms with sarcomatous stroma: molecular evidence for monoclonal origin with subsequent divergence of the epithelial and sarcomatous components. Mod Pathol.

[20] Richardson G, Robertson DI, O’Connor ME (1990). Malignant transformation occurring in mature cystic teratomas of the ovary. Can J Surg.

[21] Chan YF, Ho HC, Yau SM (1989). Ovarian mucinous tumor with mural nodules of anaplastic carcinoma. Gynecol Oncol.

[22] Nichol GE, Mills SE, Ulbright TM (1991). Spindle cell mural nodules in cystic ovarian mucinous tumors: a clinicopathologic and immunohistochemical study of five cases. Am J Surg Pathol.

[23] Fletcher CDM, Zaloudek CJ, Garg K (2013). Tumors of the female genital tract. Part A: ovary, fallopian tube, and broad and round ligament. Diagnostic Histopathology of Tumors.

